# Prenatally Detected Neonatal Neuroblastoma Associated with Achondroplasia and an SDHA Variant

**DOI:** 10.3390/neurolint18090175

**Published:** 2026-09-16

**Authors:** Penka Petleshkova, Nevena Anesteva-Ivanova, Maya Krasteva, Nikoleta Parahuleva, Atanas Ivanov, Hristo Ivanov, Anna Mihaylova

**Affiliations:** 1 Department of Obstetrics and Gynecology, Medical University of Plovdiv, 4002 Plovdiv, Bulgaria; 2 Department of Urology and General Medicine, Medical University of Plovdiv, 4002 Plovdiv, Bulgaria; 3 Department of Pediatrics and Medical Genetics, Faculty of Medicine, Medical University of Plovdiv, 4002 Plovdiv, Bulgaria; 4 Department of Healthcare Management, Faculty of Public Health, Medical University of Plovdiv, 4002 Plovdiv, Bulgaria

**Keywords:** neonatal neuroblastoma, achondroplasia, FGFR3, SDHA, next-generation sequencing, prenatal diagnosis, pediatric oncology, case report

## Abstract

**Background:** Neuroblastoma is the most common extracranial solid tumor in infancy and demonstrates marked clinical heterogeneity. To our knowledge, this is one of the very few reported cases of neonatal neuroblastoma associated with genetically confirmed achondroplasia and an additional SDHA variant. **Case Presentation:** We describe a male neonate with a prenatally detected right-sided suprarenal mass and skeletal abnormalities suggestive of achondroplasia. Postnatal evaluation was consistent with localized neuroblastoma, which was classified as low risk according to the available clinical documentation. Despite the initially favorable clinical risk profile, the tumor showed unexpected progression after initial chemotherapy, requiring treatment intensification and subsequent surgical resection. Next-generation sequencing identified a pathogenic FGFR3 c.1138G>A (p.Gly380Arg) variant confirming achondroplasia and an SDHA c.704T>C (p.Ile235Thr) variant of uncertain significance. At follow-up, the patient remained free of disease recurrence. **Conclusions:** This case expands the limited evidence on the coexistence of neonatal neuroblastoma and achondroplasia and highlights the potential value of comprehensive genomic testing in patients with atypical clinical behavior. It also demonstrates that an initially favorable clinical risk profile does not invariably predict subsequent disease behavior, emphasizing the importance of integrating clinical, radiological, and molecular findings in individualized patient management.

## 1. Introduction

Neuroblastoma is the most common extracranial solid tumor of childhood, accounting for approximately 8–10% of all pediatric malignancies. It originates from neural crest-derived cells of the sympathetic nervous system and is characterized by remarkable clinical and biological heterogeneity. While some tumors regress spontaneously, others exhibit aggressive behavior despite apparently favorable prognostic features [1,2].

Although neuroblastoma is usually diagnosed during the first years of life, prenatal and neonatal presentations are uncommon. Improvements in prenatal ultrasonography and fetal magnetic resonance imaging (MRI) have increased the detection of fetal suprarenal masses, allowing earlier diagnosis and multidisciplinary planning before delivery. Most prenatally diagnosed neuroblastomas are localized adrenal tumors with a favorable prognosis; however, occasional cases demonstrate an unexpected clinical course that cannot be fully explained by conventional biological markers [3,4].

The coexistence of neuroblastoma with congenital skeletal dysplasias is exceptionally rare. Achondroplasia, caused in most cases by pathogenic variants in the FGFR3 gene, is the most common form of disproportionate short stature but has not been considered a cancer predisposition syndrome. To date, only one case describing the coexistence of congenital neuroblastoma and genetically confirmed achondroplasia has been reported [5]. The potential contribution of additional genetic alterations, particularly variants affecting the succinate dehydrogenase (SDH) complex, remains largely unexplored. Although SDHA variants have been implicated in several hereditary tumor syndromes, their role in neuroblastoma has not yet been established [6,7].

In this report, we describe a rare genetically complex case of prenatally diagnosed neonatal neuroblastoma associated with genetically confirmed achondroplasia and an SDHA variant of uncertain significance. Beyond presenting an unusual combination of genetic findings, this case highlights the value of comprehensive genomic evaluation when the clinical behavior of a tumor does not fully correspond to its expected biological profile.

## 2. Case Presentation

This report primarily describes the prenatal and neonatal diagnostic evaluation performed by the multidisciplinary neonatology team. Information regarding the subsequent oncological management, surgery and postoperative pathological findings was obtained from the available clinical documentation and follow-up records.

A male neonate was delivered at 38 weeks of gestation by cesarean section following a pregnancy complicated by upper respiratory tract infection and elevated maternal cytomegalovirus (CMV) IgG levels. Prenatal fetal morphology assessment performed at 36 weeks of gestation revealed right-sided hydronephrosis and a rounded hyperechogenic formation located between the aorta and the renal artery in the region of the right adrenal gland. Additional prenatal findings included skeletal dysplasia characterized by shortening of the long bones below the third percentile, macrocrania, and ductal and contradural shelf of the aortic arch. Because of the suspected congenital skeletal abnormality and the presence of an adrenal mass, delivery by cesarean section was planned.

At birth, the neonate had a birth weight of 3790 g, length of 50 cm, and head circumference of 38 cm, with Apgar scores of 8 and 10 at the first and fifth minutes, respectively. Physical examination demonstrated dysmorphic features including macrocrania, hypertelorism, low-set ears, saddle-shaped nose, brachydactyly, and shortened limbs, suggestive of skeletal dysplasia. Cardiopulmonary adaptation was stable, and no major neurological abnormalities were observed during the neonatal period.

Transfontanel ultrasonography demonstrated normal lateral ventricles with prenatal periventricular hyperechogenic changes. Pediatric cardiology evaluation confirmed the presence of a fibromuscular shelf of the aortic arch without hemodynamic significance, requiring only follow-up. Initial laboratory investigations, including complete blood count, biochemical profile, electrolyte levels, and acid–base balance, were within normal neonatal limits, and maternal–fetal infection was excluded.

**Figure 1:**
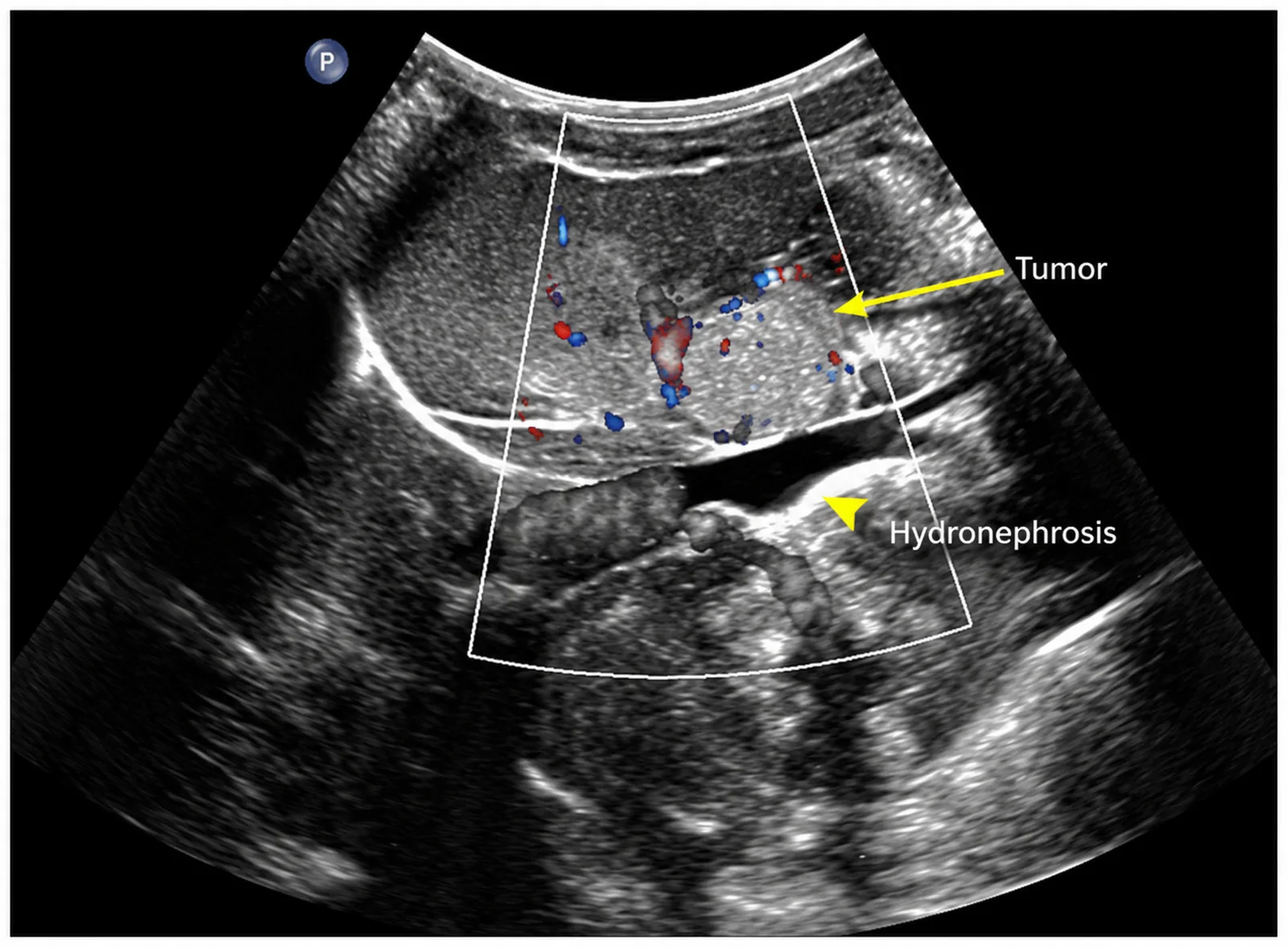
Postnatal abdominal ultrasonography demonstrating a right-sided suprarenal mass (approximately 17.7 × 14.9 mm) associated with ipsilateral hydronephrosis. The lesion is indicated by the arrow.

Postnatal abdominal ultrasonography confirmed a right-sided suprarenal mass measuring approximately 17.7 × 14.9 mm, associated with ipsilateral hydronephrosis (Figure 1). Computed tomography additionally demonstrated suspected para-aortic lymphadenomegaly. Biochemical tumor marker assessment revealed mildly elevated neuron-specific enolase (NSE) levels, while urinary homovanillic acid (HVA) and vanillylmandelic acid (VMA) remained within normal limits.

**Figure 2:**
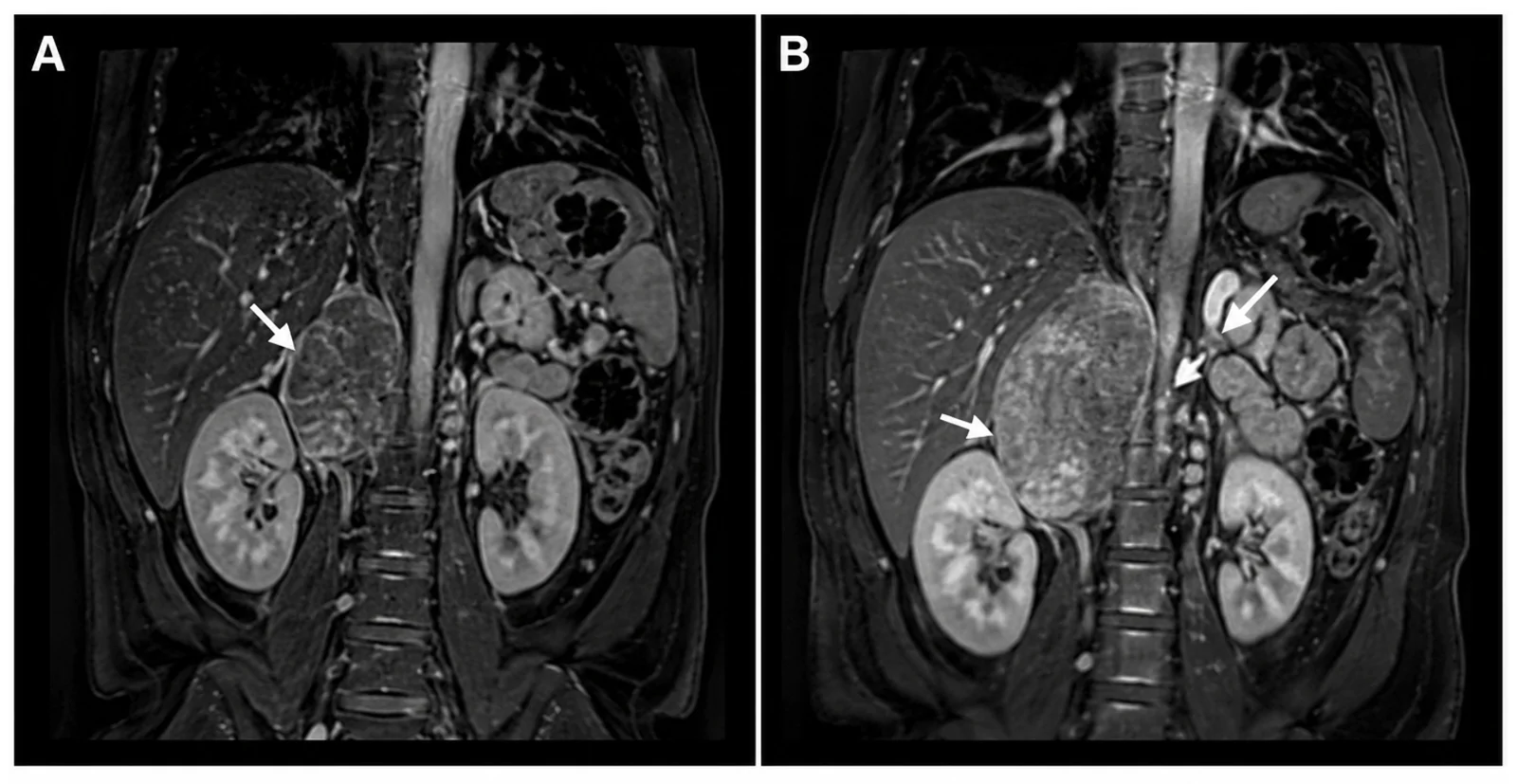
Follow-up contrast-enhanced MRI. (**A**) Right-sided retroperitoneal/paraspinal tumor in the adrenal region (arrow). (**B**) Interval tumor enlargement with regional lymphadenopathy (arrows), consistent with disease progression.

Following referral to a pediatric oncohematology department, contrast-enhanced magnetic resonance imaging (MRI) demonstrated a right-sided retroperitoneal/paraspinal low-risk neuroblastoma in the region of the right adrenal gland, causing compression of the right ureter. Despite the initially favorable clinical risk profile, the tumor demonstrated atypical clinical behavior. Initial treatment according to low-risk neuroblastoma protocols included two courses of chemotherapy. However, follow-up imaging revealed progression of the lesion with enlargement of the tumor mass and appearance of new satellite lymph nodes (Figure 2).

Due to the unexpected progression despite favorable biological characteristics, two additional chemotherapy courses were administered, resulting in substantial reduction in the tumor mass, resolution of hydronephrosis, and absence of regional lymphadenopathy. Following multidisciplinary evaluation, exploratory laparotomy with surgical excision of a small cystic tumor formation located in the right adrenal region was performed. According to the available postoperative clinical documentation, the tumor was reported as non-MYCN-amplified; however, the original laboratory methodology used for MYCN assessment was not available for retrospective verification.

### 2.1. Genetic Analysis

Given the coexistence of dysmorphic skeletal features and neuroblastoma, comprehensive genetic evaluation was undertaken. Genomic DNA extracted from peripheral blood was analyzed using a targeted next-generation sequencing (NGS) panel including genes associated with skeletal dysplasias, hereditary cancer predisposition syndromes, and developmental disorders. Variants were interpreted according to the American College of Medical Genetics and Genomics (ACMG) recommendations. Genetic analysis identified a heterozygous pathogenic FGFR3 variant c.1138G>A (p.Gly380Arg), confirming the diagnosis of achondroplasia. In addition, a heterozygous SDHA variant c.704T>C (p.Ile235Thr) was detected and classified as a variant of uncertain significance (VUS). No additional clinically significant variants relevant to the patient’s phenotype were identified. Although the precise role of SDHA alterations in neuroblastoma remains unclear, emerging evidence suggests a potential association between SDHA-related pathways and pediatric tumorigenesis.

### 2.2. Follow-Up

At follow-up, the patient remained in good general condition without evidence of residual disease or recurrence on postoperative MRI evaluation. Growth and neurodevelopment were appropriate for age, and no additional oncological complications were observed during follow-up. Long-term multidisciplinary surveillance was continued with serial abdominal ultrasonography every three months and MRI evaluation every six months due to the unusual clinical course and the identified genetically complex background (Table 1).

At the latest follow-up, the child remained clinically well, with no evidence of disease recurrence or treatment-related complications.

## 3. Discussion

Neuroblastoma is the most common extracranial solid tumor in infancy and demonstrates remarkable clinical heterogeneity, ranging from spontaneous regression to aggressive metastatic disease [1,2]. Although neuroblastoma presenting during the neonatal period is uncommon, the coexistence of neuroblastoma with genetically confirmed achondroplasia and an additional SDHA variant represents an exceptionally rare and genetically complex clinical presentation. To our knowledge, only one previous case describing the coexistence of congenital neuroblastoma and achondroplasia has been reported [5], highlighting the rarity of this association. Reports describing neonatal neuroblastoma in association with FGFR3-related skeletal dysplasia remain extremely scarce.

The identified SDHA variant remains classified as a variant of uncertain significance (VUS), and its contribution to tumor development in the present patient cannot be established. Nevertheless, alterations affecting the succinate dehydrogenase (SDH) complex have been associated with succinate accumulation, stabilization of hypoxia-inducible factors (HIFs), and activation of pseudohypoxic signaling pathways that may contribute to tumorigenesis. These mechanisms remain hypothetical in the present case and require functional validation. Although these mechanisms have been demonstrated mainly in tumors harboring pathogenic variants in other SDH complex subunits, they provide a biologically plausible framework for further investigation of SDHA-associated tumorigenesis. In the present case, functional studies evaluating SDH enzymatic activity, succinate accumulation, or tumor vascularity were not available; therefore, no causal relationship between the detected SDHA variant and the unusual clinical course can be established [6,8,9,10].

The unexpected clinical evolution observed in our patient illustrates that neonatal neuroblastoma does not always follow the course predicted by conventional biological markers. Although the tumor was classified as low risk according to the available clinical documentation, its subsequent clinical course was less favorable than initially expected [1,3]. This discrepancy reinforces the importance of combining biological, radiological, and clinical findings throughout follow-up rather than relying on a single prognostic parameter. In our patient, serial imaging played a pivotal role in identifying early disease progression and prompted timely modification of the therapeutic strategy, ultimately leading to successful disease control.

The present case is notable for several reasons. First, the tumor was detected prenatally during fetal morphology assessment, together with skeletal abnormalities suggestive of dysplasia. Advances in fetal ultrasonography and magnetic resonance imaging have made the prenatal diagnosis of neuroblastoma increasingly feasible, particularly during the third trimester of pregnancy [4,11]. Most prenatally diagnosed neuroblastomas are localized adrenal tumors with favorable biological characteristics and an excellent prognosis, although careful postnatal evaluation remains essential because individual cases may follow a less predictable clinical course [12,13,14]. In our patient, prenatal detection enabled early postnatal multidisciplinary evaluation and therapeutic planning.

Despite its favorable clinical risk profile, our patient’s clinical course differed from what would generally be expected for low-risk neuroblastoma. According to the available clinical documentation, the tumor was classified as localized low-risk neuroblastoma. Nevertheless, follow-up imaging demonstrated enlargement of the retroperitoneal mass together with the appearance of regional satellite lymph nodes [3,15]. Urinary catecholamine metabolites remained within normal limits, whereas neuron-specific enolase was only mildly elevated. Such atypical progression is uncommon in neonatal low-risk neuroblastoma and further illustrates the biological diversity of this disease [13,14,16].

The coexistence of skeletal dysplasia and neuroblastoma prompted comprehensive genetic evaluation using next-generation sequencing. The identified pathogenic FGFR3 variant, c.1138G>A (p.Gly380Arg), represents the most common molecular cause of achondroplasia [17,18,19]. FGFR3 is a key regulator of endochondral bone development and skeletal growth. Recently, [5] described a similar case of congenital neuroblastoma associated with achondroplasia. However, no additional SDHA variant was identified, making the present case genetically more complex and limiting conclusions regarding potential genotype–phenotype interactions. Although achondroplasia itself is not considered a cancer predisposition syndrome, alterations in fibroblast growth factor signaling have been implicated in several malignancies, raising interesting questions about possible shared developmental pathways.

One of the strengths of this case is the use of comprehensive genomic testing in the diagnostic work-up. In recent years, next-generation sequencing has become an increasingly valuable tool for evaluating children with congenital anomalies and rare malignancies, particularly when the clinical presentation cannot be fully explained by established diagnostic criteria. Beyond confirming the diagnosis of achondroplasia, genetic testing in our patient identified an additional SDHA variant, expanding the molecular profile of this unusual clinical presentation. Although the clinical significance of this finding remains uncertain, documenting such variants is important because accumulating evidence from carefully characterized cases may help clarify their role in disease susceptibility and phenotype variability in the future [6,7].

Our experience further highlights multidisciplinary management involving neonatologists, pediatric oncologists, radiologists, surgeons, geneticists, and pediatric cardiologists. It also emphasizes the growing role of next-generation sequencing in neonates presenting with congenital anomalies and solid tumors, particularly in cases with atypical clinical behavior or discordance between biological markers and disease progression. Early genetic evaluation may provide additional prognostic information, support long-term surveillance strategies, and contribute to the understanding of rare genotype–phenotype associations in pediatric oncology.

Taken together, this case expands the limited evidence on the coexistence of neonatal neuroblastoma and achondroplasia and illustrates how comprehensive genomic evaluation may provide clinically relevant information even when the significance of individual variants remains uncertain.

## 4. Limitations

This report primarily focuses on the prenatal and neonatal diagnostic evaluation of a rare genetically complex case. Information regarding the subsequent oncological management, surgery, and postoperative pathological findings was obtained from the available clinical documentation. Original histopathological slides, immunohistochemical findings, detailed methodological information regarding MYCN assessment, and functional studies evaluating SDH enzymatic activity, succinate accumulation, or tumor vascularity were not available for independent retrospective review. Consequently, the pathological and molecular characterization of the tumor was limited to the available clinical records, and no causal relationship between the identified SDHA variant and the unusual clinical course can be established.

Despite these limitations, the case remains clinically relevant because of the exceptionally rare association of prenatal neuroblastoma, achondroplasia caused by a pathogenic FGFR3 variant, and an SDHA variant of uncertain significance, together with its unexpected clinical progression.

## 5. Lessons Learned from This Case

This case illustrates several important clinical messages. First, prenatal identification of suprarenal masses associated with congenital anomalies should prompt early multidisciplinary evaluation. Second, discordance between favorable biological markers and clinical behavior may justify extended molecular investigation. Finally, comprehensive genomic assessment may reveal additional variants that, although not currently considered pathogenic, may contribute to improving our understanding of rare genotype–phenotype associations in pediatric oncology.

## 6. Conclusions

This case highlights the diagnostic challenges of prenatally detected neonatal neuroblastoma associated with achondroplasia and an SDHA variant of uncertain significance. The unexpected tumor progression despite the initially favorable clinical risk profile underscores the biological heterogeneity of neonatal neuroblastoma and the need for comprehensive multidisciplinary evaluation. Although a causal role of the identified SDHA variant cannot be established, this rare case adds to the limited evidence on genetically complex neonatal neuroblastoma and emphasizes the importance of integrating prenatal imaging, molecular diagnostics, and longitudinal clinical follow-up in individualized patient management.

## Figures and Tables

**Table 1:** Clinical timeline of diagnostic evaluation, treatment, and follow-up.

Timepoint	Clinical Findings/Intervention
36 weeks’ gestation	Prenatal ultrasound detected right suprarenal mass and hydronephrosis
Birth	Dysmorphic features and skeletal abnormalities identified
Neonatal evaluation	Ultrasound and CT right suprarenal mass confirmed
Initial MRI	Right-sided retroperitoneal/paraspinal neuroblastoma diagnosed
First-line therapy	Two chemotherapy courses administered
Follow-up imaging	Tumor progression and satellite lymphadenopathy observed
Additional therapy	Two additional chemotherapy courses
Surgery	Exploratory laparotomy and tumor excision
Genetic evaluation	FGFR3 pathogenic variant and SDHA VUS identified
Follow-up	No recurrence on postoperative MRI

## Data Availability

The data presented in this study are available within the article.
